# Supplementary material for: Behavioral Emotion Regulation Strategies and Symptoms of Psychological Distress Among Turkish University Students
Source: Behav Sci (Basel). 2024 Dec 26;15(1):6. doi: 10.3390/bs15010006 (PMC11761923; doi:10.3390/bs15010006)
Supplement: Supplementary file 1 [file behavsci-15-00006-s001.zip › behavsci-3283927-supplementary.pdf]

Table S1. Results of multivariate multiple regression analyses.

Model	*Wilks' Lambda*	*F*	df_1	df_2	*p*
Model 1	0.68	12.39	21	1789.5	0.001***
Model 2	0.66	10.13	27	1814.3	0.001***

Note. *** $p < 0.001$.

Table S2. Model 1 summary statistics.

Model	*R*	R^2	*SE Est.*	*F*	df_1	df_2	*p*
Depression	0.52	0.27	3.65	32.77	7	625	0.001***
Anxiety	0.45	0.20	3.26	22.07	7	625	0.001***
Stress	0.44	0.19	3.18	20.87	7	625	0.001***

Note. *** $p < 0.001$.

Table S3. Results of multiple regression analyses for Model 1.

**Model**	***B***	***SE***	**β**	***t***	***p***
**Depression**					
Intercept	2.04	2.19		0.93	0.351
Sex	-0.62	0.35	-0.06	-1.78	0.075
Age	0.00	0.08	0.00	0.05	0.959
Seeking distraction	-0.18	0.06	-0.11	-2.76	0.006**
Withdrawal	0.57	0.05	0.44	11.44	0.001***
Actively approaching	-0.19	0.06	-0.13	-3.19	0.001**
Seeking social support	0.19	0.05	0.16	4.31	0.001***
Ignoring	0.12	0.05	0.09	2.35	0.001***
**Anxiety**					
Intercept	0.81	1.95		0.41	0.679
Sex	0.38	0.31	0.05	1.24	0.217
Age	-0.02	0.07	-0.01	-0.32	0.748
Seeking distraction	-0.16	0.06	-0.12	-2.83	0.005**
Withdrawal	0.43	0.04	0.39	9.77	0.001***
Actively approaching	-0.02	0.05	-0.02	-0.44	0.633
Seeking social support	0.17	0.04	0.17	4.34	0.001***
Ignoring	0.10	0.04	0.10	2.31	0.021*
**Stress**					
Intercept	2.87	1.90		1.51	0.132
Sex	0.46	0.30	0.06	1.52	0.129
Age	-0.05	0.07	-.0.03	-0.69	0.490
Seeking distraction	-0.12	0.06	-0.09	-2.05	0.041*
Withdrawal	0.39	0.04	0.37	9.03	0.001***
Actively approaching	-0.06	0.05	-0.05	-1.09	0.274
Seeking social support	0.20	0.04	0.20	5.05	0.001***
Ignoring	0.09	0.04	0.09	2.10	0.036*

Note. Sex coded as 0 = Male, 1 = Female, $^*p < 0.05$, $^{**}p < 0.01$, $^{***}p < 0.001$.

Table S4. Model 2 summary statistics.

Model	*R*	R^2	*SE Est.*	*F*	df_1	df_2	*p*
Depression	0.53	0.28	3.64	26.45	9	623	0.001***
Anxiety	0.46	0.21	3.26	18.11	9	623	0.001***
Stress	0.44	0.20	3.18	16.83	9	623	0.001***

Note. *** $p < 0.001$.

Table S5. Results of multiple regression analyses for Model 2.

**Model**	***B***	***SE***	**β**	***t***	***p***
**Depression**					
Intercept	2.76	1.22		2.26	0.024
Sex	-0.67	0.34	-0.07	-1.93	0.054
Second-year	-0.44	0.45	-0.04	-0.97	0.332
Third-year	-1.02	0.44	-0.10	-2.33	0.020*
Fourth-year	-0.88	0.41	-0.10	-2.13	0.033*
Seeking distraction	-0.18	0.06	-0.11	-2.81	0.005*
Withdrawal	0.57	0.05	0.44	11.44	0.001*
Actively approaching	-0.18	0.06	-0.12	-3.03	0.003**
Seeking social support	0.19	0.04	0.16	4.34	0.001***
Ignoring	0.11	0.05	0.09	2.23	0.026*
**Anxiety**					
Intercept	0.74	1.09		.67	0.501
Sex	0.35	0.31	0.04	1.12	0.262
Second-year	-0.19	0.40	-0.02	-0.47	0.637
Third-year	-0.95	0.39	-0.11	-2.42	0.016*
Fourth-year	-0.61	0.37	-0.08	-1.64	0.101
Seeking distraction	-0.17	0.06	-0.12	-2.89	0.004**
Withdrawal	0.44	0.04	0.40	9.84	0.001***
Actively approaching	-0.01	0.05	-0.01	-.26	0.793
Seeking social support	0.18	0.04	0.17	4.37	0.001***
Ignoring	0.10	0.04	0.09	2.19	0.029*
**Stress**					
Intercept	1.91	1.07		1.79	0.074
Sex	0.44	0.30	0.05	1.48	0.140
Second-year	0.29	0.39	0.03	0.74	0.462
Third-year	-0.54	0.38	-0.07	-1.41	0.159
Fourth-year	-0.14	0.36	-0.02	-0.39	0.696
Seeking distraction	-0.12	0.06	-0.09	-2.10	0.036*
Withdrawal	0.40	0.04	0.37	9.11	0.001***
Actively approaching	-0.05	0.05	-0.04	-1.01	0.315
Seeking social support	0.20	0.04	0.20	5.03	0.001***
Ignoring	0.09	0.04	0.09	2.02	0.044*

Note. Sex coded as 0 = Male. 1 = Female. First-year students used as reference category in grade level. *$p < 0.05$. **$p < 0.01$. ***$p < 0.001$.
